# A Service Evaluation of the National High Secure Deaf In-Reach Service

**DOI:** 10.1192/bjo.2022.396

**Published:** 2022-06-20

**Authors:** Gurpreet Kaler

**Affiliations:** Rampton Hospital, Retford, United Kingdom

## Abstract

**Aims:**

The National High Secure Deaf Service at Rampton Hospital provides inpatient assessment, treatment and rehabilitation for D/deaf* males living with a range of difficulties including complex responses to trauma, mental health difficulties and/or learning disabilities. In 2011, the Deaf Prison In-Reach Service was established in conjunction with Yorkshire Specialist Commissioning Group and Nottinghamshire NHS Trust aiming to provide specialist support to D/deaf prisoners. * ‘D’ = Deafness as a culture, ‘d’ = deafness as a medical disability.

**Methods:**

The team evaluated the service to raise awareness of the specific needs of D/deaf prisoners by identifying and describing characteristics, demographics, trends and patterns within existing data as well as highlighting the nature of offences, prevalence of trauma and length of time over tariff. A secondary aim was to identify areas for development to adequately meet the needs of D/deaf prisoners.

**Results:**

After reviewing data for 29 prisoners (female = 3, male = 26), the most common source of support offered by the DPRIS was signposting (over 50%), followed by direct individual work (with nursing or psychology), assessment and consultancy.

Since 2011, the DPRIS has assessed 30 individuals and completed over 717 prison visits for assessments and interventions. Whilst this has been acknowledged as a small number, it has been attributed to the difficulties locating D/deaf prisoners and lack of awareness regarding the DPRIS. Currently, referrals to the DPRIS come from prison healthcare staff, but this fails to address the wider specialist needs of this population: basic communication needs, occupational needs and risk reduction work. It also excludes individuals unknown to healthcare.

Direct engagement with the DPRIS included: focused risk reduction work, anger management, mental health monitoring, and 1:1 psychology work. Prior to involvement from the DPRIS, five individuals declined to engage in prison therapy. With support from the DPRIS, two were transferred to more appropriate placements, one was recommended for transfer (not transferred) and one received mental health monitoring (nursing). One continued to decline which could be attributed to potential (lack of) motivation/readiness.

This evaluation supports the need for specialist interventions to ensure equitable access to recovery and rehabilitation.

**Conclusion:**

What Next?

It is hoped that the unique needs of this population will be communicated amongst professionals and steps will be made to address these as previously recommended in reports from the BDA (2016) and the Howard League.

